# A tracking tool for long-lasting insecticidal (mosquito) net intervention following a 2011 national distribution in Benin

**DOI:** 10.1186/1756-3305-7-6

**Published:** 2014-01-04

**Authors:** Roseric Azondekon, Virgile Gnanguenon, Frederic Oke-Agbo, Speraud Houevoessa, Michael Green, Martin Akogbeto

**Affiliations:** 1 Centre de Recherche Entomologique de Cotonou (CREC), Cotonou, Benin; 2 University of Massachusetts Amherst, Amherst, Massachusetts, USA; 3 Faculté des Sciences et Techniques de l’Université d’Abomey-Calavi, Abomey-Calavi, Benin; 4 Centers for Disease Control and Prevention, Atlanta, Georgia, USA

**Keywords:** LLIN, Monitoring, Survivorship, Fabric integrity, Gas chromatography

## Abstract

**Background:**

Following a mass distribution of long-lasting insecticidal nets (LLINs) in Benin, we used WHO guidelines to develop an assessment tool which is described in this report. It involved assessment of the three WHO indicators: survivorship, integrity and bio-efficacy.

**Methods:**

To evaluate the assessment tool, we selected four communities, two in the Southern part of the country, and two in the North. One of the two assessment communities in each geographic setting had ready access to water and a higher reported frequency of washing LLINs. It was assumed that nets in communities with greater washing frequencies would show greater loss of durability. If the tool was sensitive enough to detect such differences, the field testing would confirm its suitability for general use in different settings in Benin. While durability indicators of survival and fabric integrity were quantified using standard WHO methodology, bio-efficacy was assessed using a ‘new’ alternative (to the WHO bioassay test), involving gas chromatography. Additionally, data management used current internet technology for ‘real time’ analysis at a central monitoring location.

**Results:**

While no difference in survivorship was observed between sites with ready access to water for washing, both in the North and the South, there was a significant difference in integrity. In the South and in the North, nets from sites near water (Kessounou and Malanville) showed greater damage to integrity than did the nets from Allada and Kandi (sites far from water). As expected, LLIN integrity was significantly lower when a community was near water (p < 0.01). Bio-efficacy measurements, based on GC, were found to be so variable.

**Conclusion:**

A rapid decrease of the LLINs fabric integrity was observed in areas near water for washing following the first 6 months post-distribution. Due to the way that the insecticide is incorporated into the LLIN fiber and its migration to the surface, confounding results were observed with the GC analysis suggesting that the WHO bio-efficacy method may also be similarly affected. The report of other assessments could help to better understand the durability of the LLINs.

## Background

Malaria remains a major health issue in Sub-Saharan Africa. In addition to taking a toll in terms of health, it consumes up to 40% of public health expenditure in poor countries, an estimated cost of US$ 12 billion in lost Gross Domestic Product (GDP) every year in Africa [1]. In Benin, malaria accounts for 39.7% of health care issues. It is ranked top as one of the major diseases affecting communities [2].

Recently, it has been shown that the use of insecticide-treated materials can reduce malaria morbidity by 50 to 60% and malaria mortality by 20% [3-6]. WHO recommends that countries integrate Long Lasting Insecticide-treated Nets (LLINs) use into national plans against malaria. Among these treated materials, mosquito nets impregnated with pyrethroids are considered to be a powerful prevention tool whose mode of intervention is to break human contact with the vector [7]. LLINs provide both a physical and chemical barrier against malaria vectors [8,9]. Under epidemiological and socio-economic conditions of malaria-endemic countries, LLINs are presently the only usable method for individual and collective protection [10]. In recent years, funds allocated to the fight against malaria have significantly increased allowing considerable progress in the availability of LLINs [11,12]. At the individual level, they protect the user against mosquito bites, and at community level, they kill enough mosquitoes to reduce the number of bites in the community at large. This effect of insecticide treated nets (ITNs) on mosquito vectors may not occur unless the majority (at least 80%) of the targeted community uses them. The lethal effect of the insecticide is then reflected in younger populations of mosquitoes, and a drastic decrease of parasite transmission [13,14]. Therefore, large-scale LLINs have become a good vector control strategy in public health [15-17].

In July 2011, the Government of Benin with the support of the President’s Malaria Initiative (PMI) conducted a mass distribution campaign of Long Lasting Insecticidal Nets (LLINs). This mass distribution campaign comes to reinforce the Indoor Residual Spray (IRS) implemented in Benin since 2008. Overall, more than 4 million of LLINs were distributed across all the 77 communes of Benin. This mass distribution can significantly increase the national coverage and the use of LLINs in Benin. To maintain the impact of this vector control strategy, it is important to replace nets that do not meet WHO standards (low durability) [18,19] in a timely way such that ongoing impact is not affected.

To assess LLIN durability, WHO recommends [20] quantifying three indicators: survivorship, fabric integrity and bio-efficacy. This study describes a monitoring (tracking) tool implemented and field tested in Benin to assess the durability of the LLINs distributed. We eventually plan to use the observed rate of change in the indicators to better describe the rate at which the impact of the intervention could change. Results are discussed to inform national malaria control policies but also to show the advantages and the limitations of the new methodological approach used in this study to assess LLINs bio-efficacy.

## Methods

### Study sites

**Figure 1:**
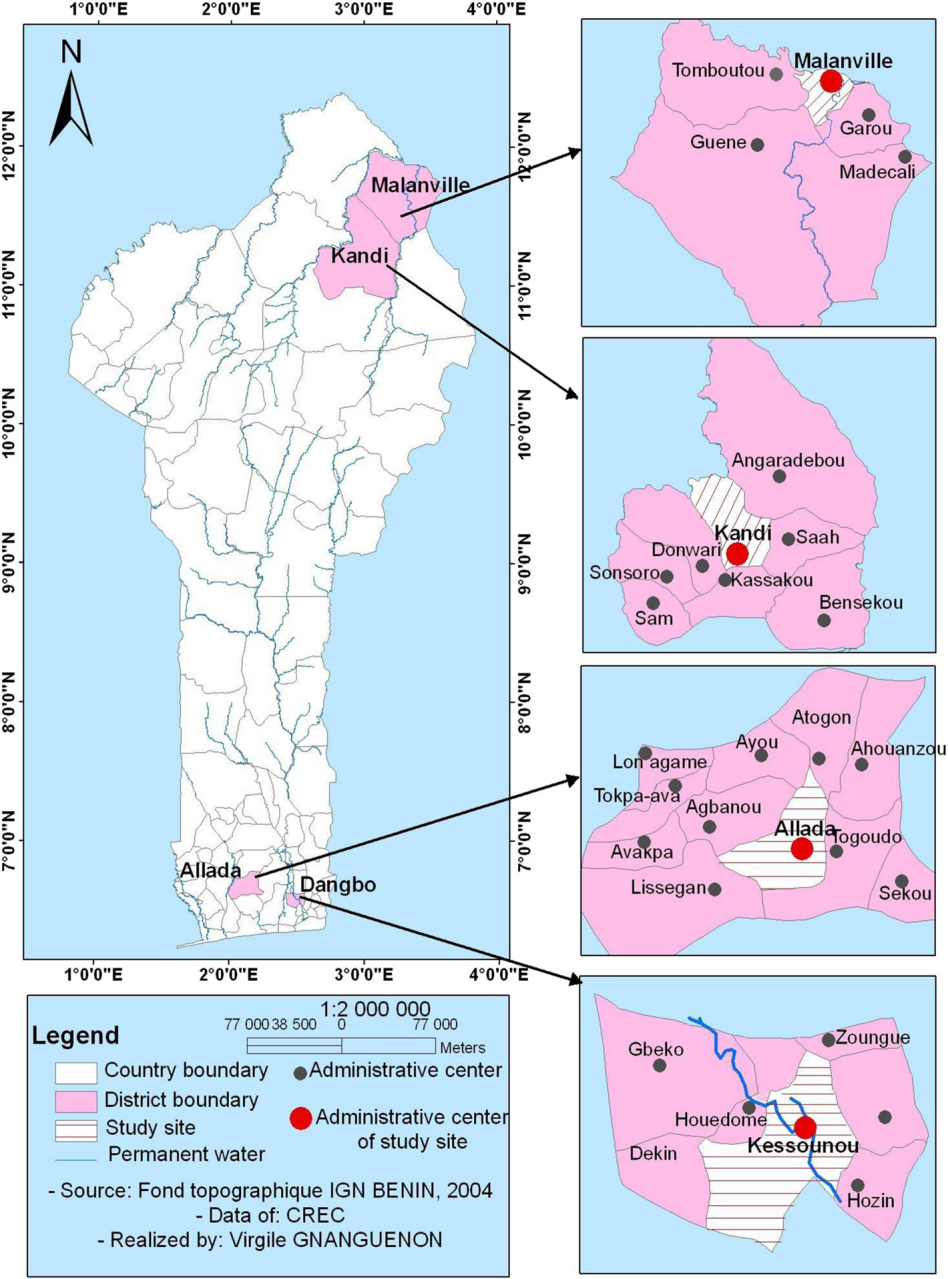
A map of Benin showing LLIN tracking sites: Kessounou and Allada in the South and Malanville and Kandi in the North.

Four arrondissements (sub districts) were selected, two in the South: Kessounou, in Oueme department and Allada, in Atlantic department, and two in the North: Kandi1 and Malanville, both in Alibori department (Figure 1). Residents of Kessounou, located on the Oueme River, have ready access to water for washing nets. In contrast, residents of Allada must carry water for washing to their homes. Similar criteria (easy access to washing site versus more difficult access) were applied to the selection of tracking sites in the North. Malanville is located near the Niger River, where water for washing nets is easily accessible. In contrast, Kandi, like Allada in the south, has a more remote water source.

To test the hypothesis that conditions on the ground affect indicators of LLIN useful life, tracking sites were intentionally selected because of ‘differences’ that would, most likely, change loss rates associated with the three indicators: (1) proximity to water for washing LLINs (expected to increase loss associated with durability and bio-efficacy by increasing the frequency of net washing); and (2) mosquito biting density/nuisance level (expected to increase loss associated with durability by increasing the frequency of LLIN use, and in turn LLIN wear and tear). Table 1 summarizes these differences.

**Table 1:** LLIN tracking sites: geographic location, climate, water for washing LLIN available/not available in the community; estimated frequency of washing*

*Based on questionnaire administered at T_***6***_	
-Water not available; estimated percentage of residents who washed the LLIN > 5 times between T_0_ and T_6_*: **≤ 0.5%** (n = 43/871)	***Allada***
	South Benin
	Climate: Guinean coastal ‘upland’ away from Oueme River
	***Kandi 1***
	North Benin
	Climate: Sudanian ‘upland’ away from Niger River
LLINs washed ‘at site’; -estimated percentage of residents who washed the LLIN >5 times between T_0_ and T_6_: **16%** (n = 157/984)	***Kessounou***
	South Benin
	Climate: Guinean coastal community on Oueme River
	***Malanville***
	Geography: North Benin
	Climate: Sudanian community on Niger River

Approximately 500 households at each site (one LLIN/household) were tracked *based on questionnaire administered to households at T_*6*_.

### Ethical consideration

This tracking study was planned under the Ministry of Health and approved by the National Ethics Committee for Health Research of the Ministry of Health of Benin. Community leaders were informed before the study and all gave consent before initiation. Written consent was also obtained from all participating households.

### LLINs used in the study

In July 2011, around four millions of Olyset® nets, a polyethylene 150D LLIN (PE-150D) impregnated with permethrin (2%), were distributed throughout the country. The monitoring tool was used at the four sites to monitor the durability of the LLIN product distributed in order to provide information for future procurement according to WHO guidelines [20].

### Household census and LLINs distribution

Before the distribution campaign, a census of all households was carried out throughout the country including our study sites. The census and the distribution process was already described by the Ministry of Health [21]. The census recorded the name of the village, the name of the head of household, the household identification number, the number of adults and children living in the house and distributed a coupon, used to obtain the LLINs. The information was recorded in a master list. Net allocation to the household was based on the household size and the ratio one net for two persons (the national policy for universal coverage). This distribution campaign covered an average of 90% of the households in the country.

LLINs distributed have labels sewn into them at factory that helps to distinguish them from those distributed during other campaigns or received from other sources.

### Households sampling and bar coding of LLINs (T0)

WHO suggested estimating sample size on the basis of the attrition of the LLIN product. But in Benin, PE-150D product attrition rate is not known. Sample size was then estimated following the WHO guidelines, which reported that a sample of 250 LLINs will allow detection of a 10% point difference if the best-performing product has an attrition rate of 10% and a 12 point difference with an attrition rate of 20% [20]. This sample size was doubled and 500 LLINs were selected by study sites to provide more precision in point difference detection. A subsample of 50 LLINs representing 10% of the total sample was randomly selected to measure insecticidal activity.

One LLIN per household was selected at each study site. Household selection at each of the four sites took into account the number of villages to ensure a representative sampling of the study site. For example, Kessounou is divided in five villages: Kodonou, Kessounou, Glehoue, Hetin-Sota and Glahounsa. Because the assessment was based on information from 500 LLINs, approximately 100 households (LLINs) were selected at random in each of the five villages. Table 2 summarizes the selection process. Households included in each village were randomly selected from the registration list of the distribution.

**Table 2:** Distribution of tracking households by location

**District**	**Sector**	**Code**	**Households selected (T**_**0**_**)**	**Households visited (T**_**6**_**)**	**% completion**
**Kessounou**					
	Kodonou	KOD	100	98	98
	Kessounou	KES	100	99	99
	Glehoue	GLH	100	98	98
	Hetin-Sota	HS/HSZ	100	99	99
	Glahounsa	GLA	100	99	99
	**Total**		**500**	**493**	**99**
**Allada**					
	Alomey-Ahito	ALO/AHI	100	82	82
	Gbowele-Dodomey	GBO/DOD	100	84	84
	Dogoudo-Gbegamey	DOG/GBE	100	84	84
	Donou-Togoh	DON/TOG	100	80	80
	Tokpota-Zebou	TOK/ZEB	100	90	90
	**Total**		**500**	**420**	**84**
**Kandi 1**					
	Damadi	DAM	100	87	87
	Gansosso	GNS	100	85	85
	Keferi	KEF	100	95	95
	Pede	PED	100	86	86
	Gandokossikana	KSK	100	98	98
	**Total**		**500**	**451**	**90**
**Malanville**					
	Wouro-yesso	WOY	100	90	90
	Kotchi1	KOT	100	95	95
	Kotchi2	KCH	100	92	92
	Haro-banda	GAL	101	90	90
	Galiel	GLL	100	88	88
	**Total**		**501**	**455**	**91**
	**TOTAL**		**2000**	**1819**	**91**

Two teams composed of two technicians and a local village health worker (VHW) visited each selected household. The head of household or an adult person acting on behalf of the head was interviewed. In certain occasions where no appropriate respondent was found in a particular household, the visit of the next home was scheduled. Approximately 500 households (500–501), where campaign net(s) had been hung (and were in use), were selected at each tracking site. The teams identified the campaign LLIN (on the basis of the label sewn) in each selected household (or randomly chose one net, if multiple nets were present). They marked the LLIN for tracking assessment using a double marking system, an additional label with a unique study code plus an indelible ink mark. The team also recorded the GPS coordinates of each selected household for follow up visits. Google Earth 6.1 was used to map the study households. Written consent, to return at 6-monthly intervals, was obtained from the head of household or an adult living in the household.

### Prospective survey and questionnaire

Households included for follow-ups were located by the name of the head of household and by GPS coordinates. Households that were not opened for inspection after two visits at the 6 months assessment visit were visited at the following assessment visits until they are recorded as unresponsive for three assessment visits and replaced.

A questionnaire was used to collect data from the head of household or an adult living in the household. The collected information included the status of each LLIN, the pattern of LLIN use and handling, observations on fabric integrity and the condition of the LLIN.

GPS coordinates and net codes were entered into the database. In the field, data were recorded on PDAs (Samsung Galaxy Tablets).

### Measurement of indicators of LLINs durability

#### Survivorship

Survivorship at T0 (enrollment visit) was 100% (attrition was 0%). After six months, each tracking household received a follow-up visit. A visual verification of the presence/absence of the coded LLIN was done and when the coded LLIN was not in the house, the assessment teams determined how it was lost. Households that were not opened for assessment were re-visited and if it was still closed it was targeted for re-visit in the next 6 months assessment visit.

### LLINs fabric integrity

At the enrollment visit (T0), none of the LLINs had holes (loss of fabric integrity was 0%). After 6 months, LLIN fabric integrity was assessed by a visual examination, without removal of coded nets from tracking households. LLIN physical integrity was assessed by verifying if the coded LLINs had holes or not and recorded the major holes category found on the LLINs as follow:

smaller than a thumb (0.5–2 cm),

larger than a thumb but smaller than a fist (2–10 cm) and

larger than a fist but smaller than a head (10–25 cm)

Holes less than 0.5 cm were ignored. The 6 months follow up assessment only recorded the major categories (no holes size-4 were counted) of holes found in the LLINs but did not count them. Their natures, locations, evidence of repairs and the type of repair were also not recorded, and represented an important limitation of the 6 months assessment study.

### Bio-efficacy assessment method using gas chromatography (GC)

WHO recommends the use of the cone bio-assay method [20] for monitoring bio-efficacy. However, problems related to rearing the large numbers of colonized, pyrethroid-susceptible vector females, needed to support the application of this method to a statistically meaningful number of LLINs, hinder its correct use. An alternative method, the colorimetric test was developed for use with deltamethrin-treated LLINs [22,23]. Colorimetric assessment of LLIN bio-efficacy involves a two-step process. In step one, a magnetic sampling device (MSD) is used to sample the insecticide level on the LLIN surface (without removing or damaging the LLIN). The amount of insecticide on the MSD sample, a filter paper disc, is proportional to the amount of insecticide on the LLIN. Because sampling is standardized, results for different LLINs in the sampling frame are comparable. The second colorimetric assessment step uses colorimetric chemistry to estimate the amount of deltamethrin in the sample. Colorimetric results have been validated, by comparing WHO cone test bio-assay results and colorimetric results for a series of LLINs with different surface levels of deltamethrin. Using the standard curve from this study, it is possible to interpret the colorimetric result in terms of whether or not an LLIN meets the minimum WHO ‘threshold’ for bio-efficacy (nets causing >80% mortality in a cone bio-assay test).

**Figure 2:**
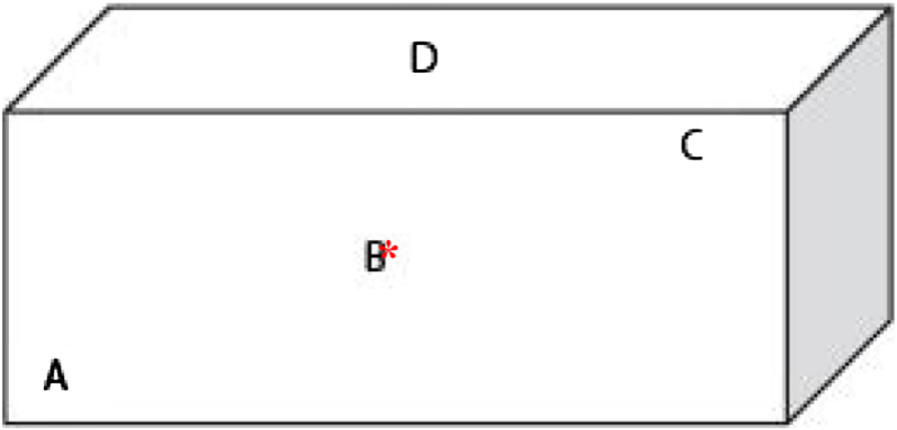
Identification of sampling positions.

A conceptually similar ‘chemical test’ approach was used for tracking the bio-efficacy of the PE-150D LLINs in this assessment. The approach, based on gas chromatography (GC), was developed in order to circumvent the problems previously described that are associated with the WHO cone bio-assay method for bio-efficacy assessment. With PE-150D technology, the insecticide molecules migrate to the surface continuously replacing lost insecticide at the surface, where vectors are exposed to its effects. Based on this theory, the LLIN surface insecticide level sampling tool was modified to enable sampling of PE-150D LLINs without removal or replacement; samples were collected on position B (Figure 2). This was done as a precautionary measure since the owners may put blankets on top of position D of the net which may rub some of the insecticide off. Also, placement on Position A under the mattress may result in the removal of the insecticide. Therefore, the best position to collect the sample is position B in the middle of the net.

**Figure 3:**
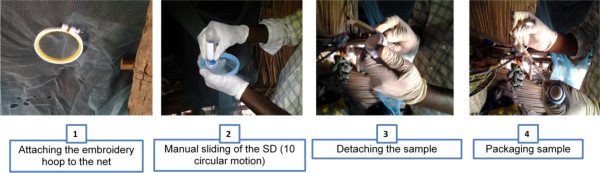
Sampling a mosquito net at Kessounou.

The net to be tested is hung and position B is identified and fixed on an embroidery hoop (4 inches diameter). A portion of lens paper is applied to the surface of a cap attached to a 50-mL plastic tube. The lens paper is rubbed along the inside diameter of the hoop for 10 rotations. The lens paper (25 mm diameter) is removed and inserted into a 1-mL syringe and compressed with the plunger. The reverse side of the net is repeated in the same manner (Figure 3). 100 μL of acetone containing 0.05 mg/mL of triphenyl phosphate as an internal standard is added to the syringe containing the 2 compressed portions of the lens paper. The syringe outlet is plugged to allow the paper to soak for 5 minutes. After removal of the plug, the acetone is eluted via the syringe plunger into a small tube followed by another 100 μL of acetone. 1 μL of the eluant is injected into the GC (SRI 8610C GAS CHROMATOGRAPH) and the permethrin and internal standard is detected using flame ionization detection with hydrogen as the carrier gas. Sample response is compared with the response from a calibration standard and the amount of permethrin adhering to the lens paper is determined.

To estimate a bio-efficacy threshold for the GC method, 37 PED-150 LLINs (11 new nets and 26 nets that were in use in field for 3 to 4 months) were submitted to Cone test bioassay following WHO guidelines [24]. Interpretation of results (how GC test data are used to predict whether or not an LLIN would meet the minimum WHO bio-efficacy threshold) was done based on the comparison approach, outlined for the colorimetric method. A cut off value, established by comparing GC results with WHO cone test bio-assay results for the same LLINs, some of which met or exceeded the WHO minimum threshold for bio-efficacy and some which did not, was used to interpret the GC results. Receiver Operating Characteristics (ROC) analysis, applied to the two data sets, GC and WHO cone bio-assay, was used to define the cut off value for nets that would be expected to meet the WHO cone test threshold. A minimum GC permethrin concentration was identified and used as a cut off value to estimate the percentage of nets at T0 and T6 that would not meet WHO minimum standards for bio-efficacy.

### Quality of the data

The questionnaire form used in the study was created with ODK Collect 1.2.2 which allows easy data collection on Samsung Galaxy Tab 10.1 used as data terminals. At the end of each assessment visit day, data collected were directly uploaded to a cloud server, and retrieved at the end of the assessment for analysis.

Approximately 10% of the households were revisited independently to make sure that the survey was not biased.

### Data analysis

#### Survivorship and attrition

The number of coded LLINs in the location, the proportion of the indicator and 95% confidence interval was reported.

The indicators were estimated as follow:

Survivorship:

$$\frac{\mathrm{Total}\;\mathrm{coded}\;\mathrm{LLINs}\;\mathrm{still}\;\mathrm{present}\;\mathrm{in}\;\mathrm{the}\;\mathrm{households}\;\mathrm{selected}\;}{\mathrm{Total}\;\mathrm{coded}\;\mathrm{LLINs}\;\mathrm{at}\;\mathrm{the}\;\mathrm{enrollment}\;\left(T_{0}\right)}\times 100$$

Attrition rate-1 for nets that have been destroyed or disposed of (Physical damage):

$$\frac{\mathrm{Total}\;\mathrm{number}\;\mathrm{of}\;\mathrm{coded}\;\mathrm{LLIN}\;\mathrm{reported}\;\mathrm{as}\;\mathrm{thrown}\;\mathrm{out}\;\mathrm{due}\;\mathrm{to}\;\mathrm{wear}\;\mathrm{and}\;\mathrm{tear}\;\mathrm{in}\;\mathrm{surveyed}\;\mathrm{households}\;}{\mathrm{Total}\;\mathrm{coded}\;\mathrm{LLINs}\;\mathrm{at}\;\mathrm{enrollment}\;\left(T_{0}\right)}\times 100$$

Attrition rate-2 for nets not available for sleeping under (Removal):

$$\frac{\mathrm{Total}\;\mathrm{number}\;\mathrm{of}\;\mathrm{coded}\;\mathrm{LLIN}\;\mathrm{reported}\;\mathrm{as}\;\mathrm{given}\;\mathrm{away},\mathrm{stolen},\mathrm{sold}\;\mathrm{or}\;\mathrm{used}\;\mathrm{in}\;\mathrm{another}\;\mathrm{location}\;}{\mathrm{Total}\;\mathrm{coded}\;\mathrm{LLINs}\;\mathrm{at}\;\mathrm{enrollment}\;\left(T_{0}\right)}\times 100$$

Attrition rate-3 for nets re-purposed (Re-purposed):

$$\frac{\mathrm{Total}\;\mathrm{number}\;\mathrm{of}\;\mathrm{coded}\;\mathrm{LLIN}\;\mathrm{reported}\;\mathrm{as}\;\mathrm{being}\;\mathrm{used}\;\mathrm{for}\;\mathrm{another}\;\mathrm{purpose}\;\mathrm{in}\;\mathrm{surveyed}\;\mathrm{households}\;}{\mathrm{Total}\;\mathrm{coded}\;\mathrm{LLINs}\;\mathrm{at}\;\mathrm{enrollment}\;\left(T_{0}\right)}\times 100$$

The survivorship rate plus attrition rate-1, attrition rate-2 and attrition rate-3 was added up to 100%.

Two locations were reported to show significantly different survivorship at the 6 months assessment visit if the 95% confidence limits for survivorship do not overlap.

#### Fabric integrity

Physical integrity was analyzed for all the coded LLINs found and assessed in the households (and used for sleeping under). One WHO indicator was calculated at the 6 months assessment visit: the proportion of LLINs with holes.

Proportion of LLINs with any holes (with 95% confidence interval):

$$\frac{\mathrm{Total}\;\mathrm{number}\;\mathrm{of}\;\mathrm{coded}\;\mathrm{LLIN}\;\mathrm{with}\;\mathrm{at}\;\mathrm{least}\;\mathrm{one}\;\mathrm{hole}\;\mathrm{of}\;\mathrm{size}\;1‒3\;}{\mathrm{Total}\;\mathrm{number}\;\mathrm{of}\;\mathrm{coded}\;\mathrm{LLINs}\;\mathrm{found}\;\mathrm{and}\;\mathrm{assessed}\;\mathrm{in}\;\mathrm{surveyed}\;\mathrm{household}}\times 100$$

Holes index of the LLINs was not calculated due to the limit of 6 months questionnaire that did not count each category of holes.

### Bio-efficacy

For assessment of bio-efficacy, a sub-sample (n = 50/site) of LLINs was randomly selected and tested to determine the amount of insecticide on the surface of the net. The percentage of nets in the sample falling below a cut-off value for LLINs that cause > 80% mortality in a WHO cone bio-assay test [24] was used to quantify net loss associated with bio-efficacy at T6.

### Factors associated with durability of the LLIN

Other factors that contributed to the LLINs durability, as measured by fabric integrity and insecticidal activity, were assessed by multivariate regression analysis. The contributing factors included the house environment and behaviour related to net use, handling and washing, was derived from answers to the questionnaire used for the sub-sample of 50 LLINs selected for bio- efficacy in each location (around 200 LLINs in total).

## Results

### Survivorship and attrition

Table 3 summarizes T6 results for survivorship and attrition. One hundred forty seven out of 2002 coded LLINs were no longer present in the households where they were located at T0. That is, after 6 months, survivorship had dropped from 100% to between 90 and 96%, giving attrition of 10%, 9%, 4% and 6% percent respectively in Kessounou, Allada, Kandi and Malanville. Attrition rate-2 (LLINs removal) was the principal reason for LLINs lost; ranging from 4-8%. Attrition rate-1 was the second cause of LLINs lost; ranging from 0-2%. Attrition rate-3 was very low and only observed at Kessounou (Table 3). We did not assess factors associated with higher and lower survivorship loss rates, however, differences in survival between locations in the South and the North were significant (p < 0.05).

**Table 3:** **LLIN tracking T**_**6 **_**results by study sites: survivorship and attrition**

		**Kessounou**	**Allada**	**Kandi**	**Malanville**
**Survivorship**					
Total coded LLINs (T_0_)	N	501	500	500	501
Visited households (T_6_)	N	493	420	451	455
People covered	N	1518	971	1201	1243
Average people per LLINs	N	3.08	2.31	2.66	2.73
LLINs missing	N	49	46	21	31
**Survivorship**	%	90	91	96	94
**CI 95%**		87.30-92.52	87.95-93.03	93.66-97.24	91.35-95.61
**Attrition**					
(LLINs missing)	N	(49)	(46)	(21)	(31)
‘Physical damage’ responses	N	10	5	1	3
‘Removal’ responses	N	35	41	20	28
‘Re-purposed’ responses	N	4	0	0	0
***Attrition rate-1***	(%)	2	1	0	1
***CI 95%***		01.09-03.63	00.43-02.32	00.04-01.12	00.20-01.75
***Attrition rate-2***	(%)	7	08	4	5
***CI 95%***		05.07-09.56	06.10-10.94	02.60-06.10	03.89-07.96
***Attrition rate-3***	(%)	1	0	0	0
***CI 95%***		00.31-02.03			
**Total attrition**	(%)	10	9	4	6
**CI 95%**		07.48-12.70	06.97-12.05	02.76-06.34	04.39-08.65

### LLINs fabric integrity

At the 6 months assessment visit, LLINs found with holes (Table 4) was 36% at locations with less access to water for washing and 52-64% at locations with ready access to water (p < 0.01). The number of LLINs with size 1 holes was relatively low at all sites (when compared with the number of LLINs observed with size 2 and 3 holes).

**Table 4:** LLIN found with holes

		**Kessounou**	**Allada**	**Kandi**	**Malanville**
LLINs found and assessed	N	444	374	430	424
LLINs with any hole(s) (T_6_)	N	230	134	153	271
	%	52*	36	36	64**
	IC95	[45.04-56.54]	[28.96-40.92]	[31.05-40.31]	[59.14-68.49]
LLINs with size 1 holes	N	12	12	24	34
	%	5	9	16	13
	IC95	[2.72-8.94]	[4.71-15.12]	[10.32-22.44]	[08.85-17.09]
LLINs with size 2 holes	N	103	52	55	120
	%	45	39	36	44
	IC95	[38.24-51.46]	[30.51-47.6]	[28.36-44.09]	[38.27-50.42]
LLINs with size 3 holes	N	115	70	74	117
	%	50	52	48	43
	IC95	[43.36-56.65]	[43.44-60.94]	[40.22-56.58]	[37.19-49.30]

*p < 0.05, **p < 0.01.

### Assessment of bio-efficacy

#### Determining a GC threshold for LLINs that meet minimum WHO bio-efficacy criteria

**Figure 4:**
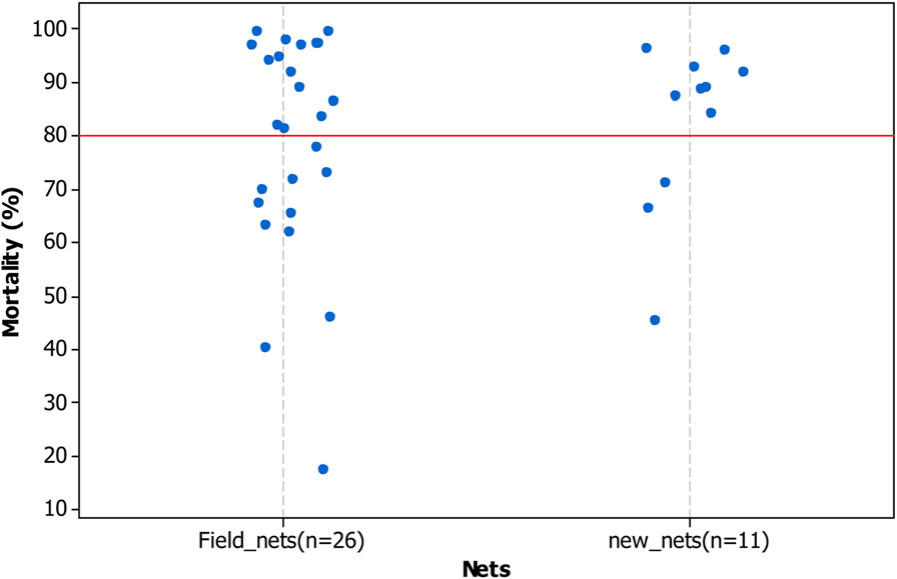
Mortality observed with WHO cone test and used to determine GC threshold.

**Figure 5:**
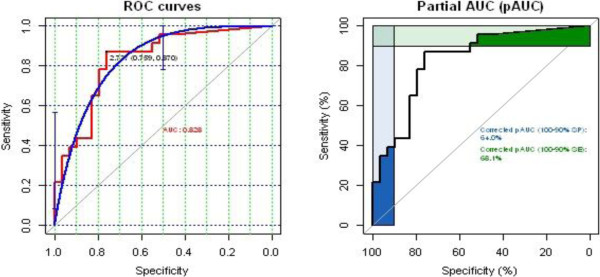
ROC curves showing prediction of WHO cone bio-essay results by GC method.

Figure 4 showed the mortalities observed with WHO cone test bioassay. On the 37 LLINs, 23 have mortality >80%. The mean mortality observed was 83% for the new nets and 79% for the nets collected from field. The minimum mortality was 18% mortality and the maximum was 100%. The median mortality was 89% for the new nets and 83% for the net selected from the field. Comparing the results with the GC method, we identified a GC value of 2.73 μg/sample or greater, for identification of LLINs that would be expected to cause >80% mortality in a WHO cone bio-assay. That is LLINs with permethrin levels high enough to meet the WHO minimum threshold for adequate LLIN bio- efficacy. LLINs giving GC results less than the 2.73 μg cut off were counted as not meeting the minimum WHO threshold for bio-efficacy. Statistical evaluation of the test, using Receiver Operating Characteristics (ROC) analysis showed that the GC method can predict the WHO result within reasonable margins (specificity = 75.9%, sensitivity = 87.0%, AUC = 0.83 (0.7-0.92)) (Figure 5). The sampling frame for bio-efficacy testing was determined to be 172 assessment LLINs, selected at random (*n.b* analysis of this many nets by means of the WHO cone bio-assay would be logistically very difficult). Despite the need for additional development work, the GC approach was used in the tracking study.

### Bio-efficacy of LLINs 6 months post-distribution

**Figure 6:**
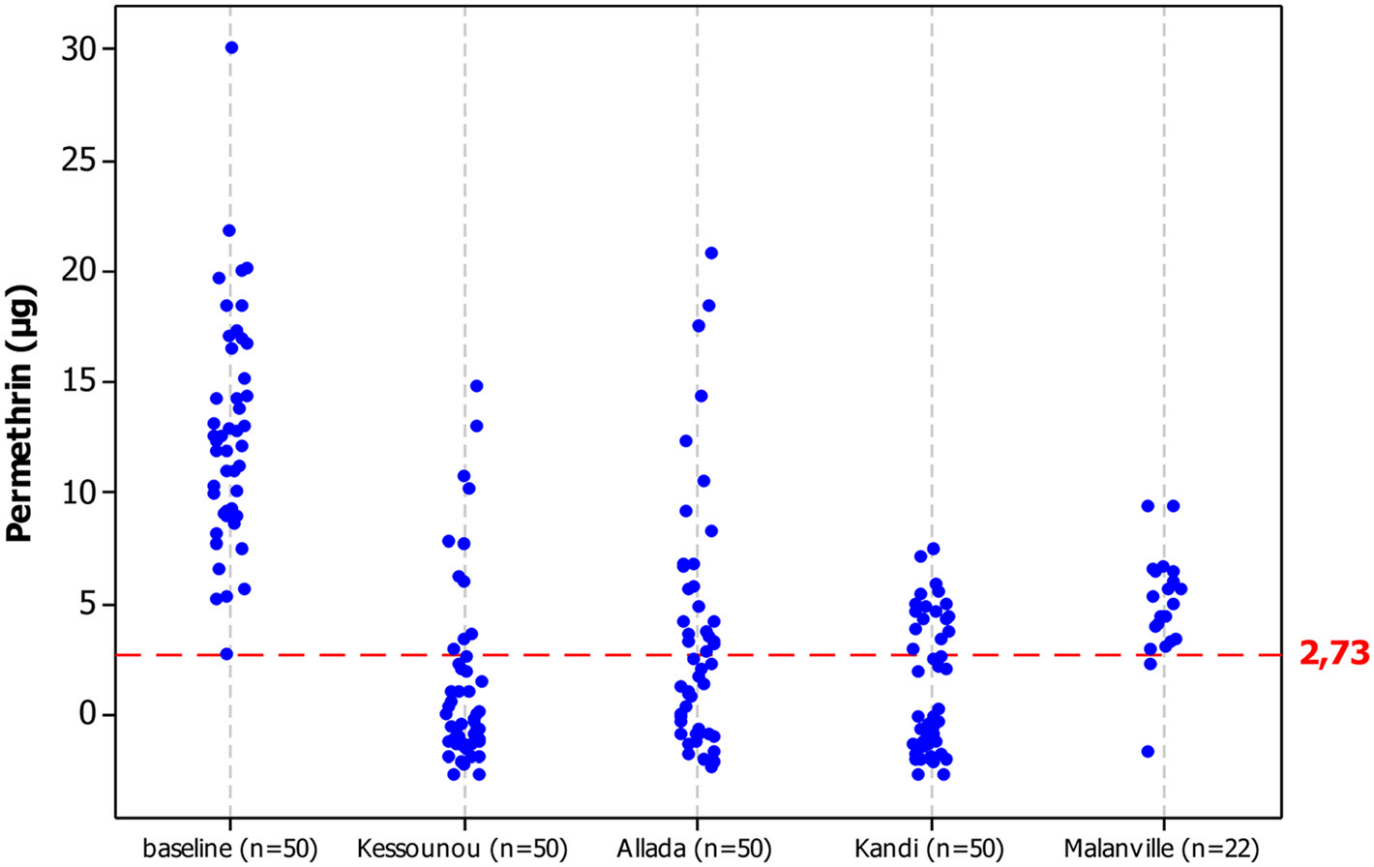
Scatter plot of LLIN surface insecticide (permethrin) levels for 212 LLINs.

**Figure 7:**
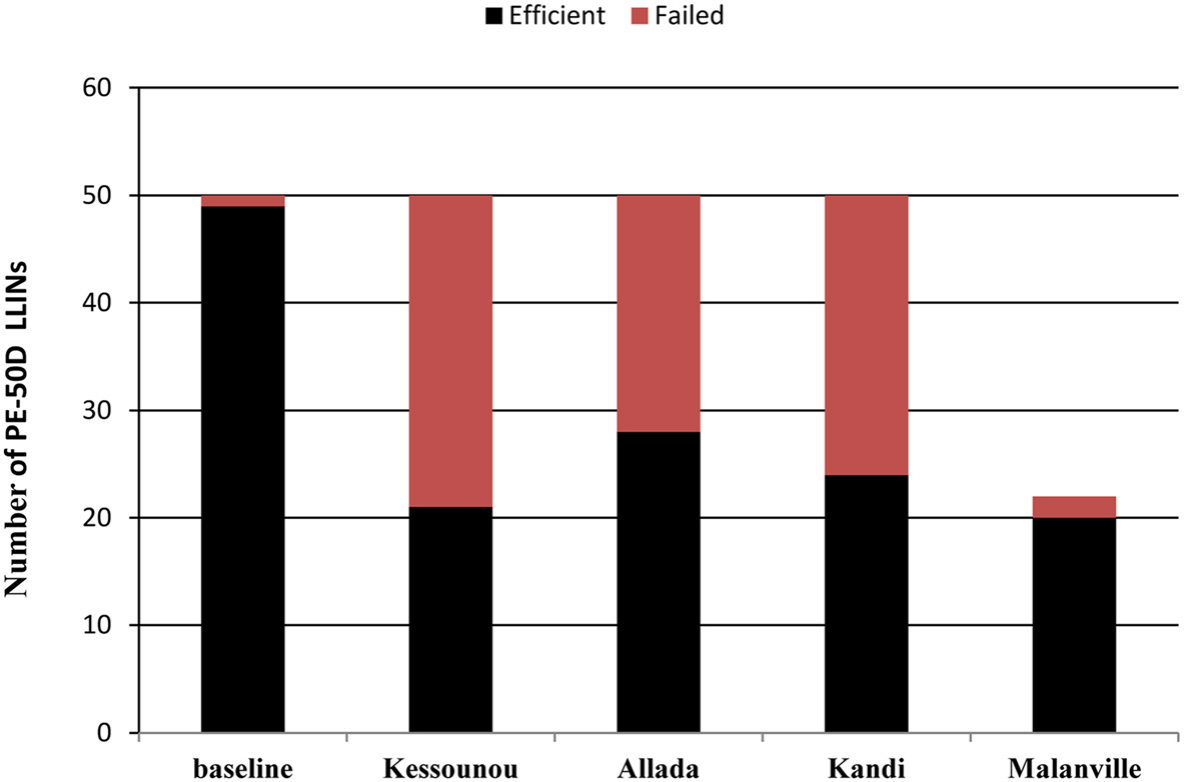
**Net loss associated with bio-efficacy.** Number of nets with insecticide levels below and above WHO threshold T0 versus T6.

A total of 222 nets were subjected to GC analysis to assess bio-efficacy. Results are summarized in Figures 6 and 7. There was significant loss associated with bio-efficacy at all four sites during the assessment period (T0-T6) (p = 0.0001). However, there was no significant difference between locations with more versus less access to water for washing. Measurements were based on gas chromatographic analysis of LLIN surface samples collected at randomly selected households in each study at T6. Results are compared with baseline (T0) measurements on recently distributed nets using the same approach. The red dotted line shows the GC cut-off equivalent to a surface insecticide level high enough to give >80% mortality in the WHO cone bio-assay, a minimum threshold for adequate LLIN bio-efficacy set by WHO.

Bio-efficacy at baseline (T0) was high. The results showed that 98% (49/50) had surface insecticide levels that were above the minimum WHO threshold for bio-efficacy (loss associated with bio-efficacy at T0 was set at 2%). After six months of use, net loss associated with bio-efficacy was 58% in Kessounou, 52% in Kandi, 44% in Allada and 9% in Malanville. At T6 there were 42% LLINs below the GC cut off for WHO minimum bio-efficacy.

### Factors associated with LLINs durability

Table 5 indicated responses from a questionnaire used to assess the house environment and behaviour related to net use, handling and washing of the sub-sample selected. At Kessounou and Malanville, most residents indicated that they washed their nets between 2–5 times in the preceding 6 months, whereas most residents at Allada and Kandi, stated that washing occurred less frequently <2 times in the preceding 6 months. Modalities with very few responses were aggregated for the multivariate analysis.

**Table 5:** Responses from the questionnaire administered to the sub-sample selected in each site

**Predictors**	**Modalities**	**KESSOUNOU**	**ALLADA**	**KANDI**	**MALANVILLE**
**Washing frequency**	None	7	21	21	10
	1 time	8	21	18	7
	2-5 time	25	7	11	26
	6-10 time	2	0	0	5
	10+	7	1	0	2
**Maintenance**	Good	18	19	19	17
	Low	31	31	31	33
**LLINs use**	Don’t know	0	1	0	0
	Not use	0	2	0	1
	Often	5	10	7	4
	Every night	44	37	43	45
**Roof of the house**	Pave stone	2	0	0	3
	Straw	15	0	1	9
	Sheet-metal	31	50	48	38
	Tile	1	0	1	0
**LLINs position**	Hanged	44	37	41	35
	Stiked	1	7	9	14
	Tidy	4	6	0	1
**Location of the kitchen of the house**	Outside	35	48	35	46
	Inside	14	2	14	4
**Fabric integrity of the LLINs**	With holes	14	35	30	13
	Without holes	35	15	20	37
**Distance to water for washing**	>5 km	0	50	50	0
	< 0.5 km	49	0	0	50

In summary (Table 6), the 6 months data showed that frequency of LLINs use, sleeping material, washing frequency and the distance to water for washing were significantly associated with loss of fabric integrity (p < 0.05).

**Table 6:** Factor associated with loss of fabric integrity

**Factors**	**Modalities**	**LLINs with any holes (N)**	**Total LLINs**	**% of LLINs with any holes**	**OR**	**OR (95%CI)**	**P (Wald test)**	**P (LR-test)**
**Frequency of LLIN use**	Often	7	30	23.33	1.00	-	-	0.004
	Every night	100	169	59.17	4.76	[1.94-11.71]	0.006	
**Sleeping material**	Bed	34	86	39.53	1.00	-	-	0.016
	Mat	72	111	64.86	2.82	[1.58-5.05]	0.018	
**Washing frequency**	None	20	59	33.90	1.00	-	-	0.001
	1	21	54	38.89	1.24	[0.58-2.67]	0.860	
	2-5	66	86	76.74	6.43	[3.08-13.43]	0.003	
**Location of the Kitchen**	Outside	93	176	52.84	1.00	-	-	0.369
	Inside	14	23	60.87	1.39	[0.57-3.37]	0.366	
**LLINs maintenance**	Good	37	73	50.68	1.00	-	-	0.976
	Low	70	126	55.56	1.22	[0.68-2.17]	0.976	
**Distance to water for washing**	>5 km	35	100	35.00	1.00	-	-	0.020
	< 0.5 km	72	99	72.73	4.95	[2.71-9.06]	0.020	

Table 7 showed that only factors like frequency of LLIN use and sleeping material were significantly associated with insecticide decay (p < 0.05). Factors like washing frequency, LLINs maintenance, location of the kitchen and distance to water for washing did not seem to play a key role in the insecticide decay observed.

**Table 7:** Factors associated with loss of insecticide bio-efficacy

**Factors**	**Modalities**	**N ineffective**	**Total**	**% ineffective**	**OR**	**OR (95%CI)**	**P (Wald test)**	**P (LR-test)**
**Frequency of LLIN use**	Often	11	28	39.29	1.00	-	-	0.028
	Every night	89	144	61.81	2.50	[1.09-5.73]	0.031	
**Sleeping material**	Bed	65	104	62.50	1.00	-	-	0.0117
	Mat	35	68	51.47	0.64	[0.34-1.17]	0.12	
**Washing frequency**	None	31	53	58.49	1.00	-	-	0.728
	1	28	50	56.00	0.90	[0.41-1.97]	0.431	
	2-5	41	69	59.42	1.04	[0.50-2.15]	0.751	
**Location of the Kitchen**	Outside	86	151	56.95	1.00	-	-	0.451
	Inside	14	021	66.67	1.51	[0.58-3.96]	0.455	
**LLINs maintenance**	Good	33	064	51.56	1.00	-	-	0.489
	Low	67	108	62.04	1.54	[0.82-2.87]	0.489	
**Distance to water for washing**	>5 km	59	100	59.00	1.00	-	-	0.17
	<0.5 km	41	072	56.94	0.92	[0.5-1.7]	0.174	

## Discussion

The National Malaria Control Program of Benin conducted a country-wide distribution of permethrin-treated Olyset® LLINs in July 2011. As early as 6 months into the assessment, we observed that measures of fabric integrity and bio-efficacy had decreased rapidly bringing into question the assumption that LLIN condition remains “relatively uniform for at least three years”. The report of other assessments will help us to more understand the effective life of the intervention. However, loss associated with bio-efficacy and fabric integrity during the first six months, was great enough to suggest that the impact of the LLIN intervention on malaria transmission could be affected.

After the first 6 months, survivorship has decreased to 93%. The main cause of net loss or missing nets was due to the removal of nets. Net missing due to poor physical condition was low. These findings suggest that LLIN survivorship would drop significantly during years two and three post distribution if the attrition rate observed continue at the same rate and could affect the impact of the LLIN intervention on malaria transmission.

A high-coverage and compliance with nightly use of LLINs, provides a ‘community protection’ benefit. Considering the observed attrition rate, over six months, it may well be that, at best; any ‘community’ protection associated with the intervention would be higher in some areas and could disappear in other areas within two years or less if the LLINs were removed and used in areas different to their initial locations and net loss associated with poor physical condition increased. Additionally, there was anecdotal evidence that homeowner re-purpose LLINs in ways that increase the attrition rate. In Kessounou, for example, a striking cause of the loss included LLINs that had been converted into fishing nets, as well as covers for protecting crops and animals. Strengthening information and communication activities around future distribution campaigns should be evaluated and incorporated into distribution campaign strategy to increase the value that community members place of correct use of nets [25].

While there were differences in survivorship, associated with geo-climatic variation (South versus North), the differences in fabric integrity, seen at geo-climatically similar sites where access to water was different, were more pronounced. LLINs found with any holes were greater in areas where water (for washing nets) was easily obtained. Proximity to a LLIN washing site seems to encourage more frequent washing of nets, thereby accelerating loss of fabric integrity. The effect of local differences on LLIN physical integrity loss, such as proximity to water, as well as regional differences, e.g. seasonal rainfall patterns, point out the importance of conducting tracking net interventions in different geographic areas [26]. Frequent washing of nets, >5 times in 6 months, was 3–4 times more common in river sites of Kessounou and Malanville, than in upland sites of Allada and Kandi, where water for washing was transported by community members. This factor as well as the sleeping material and the frequency of LLIN use played a significant role in loss associated with LLIN integrity.

Loss associated with fabric integrity increase the probability of man-mosquito contact and transmission of malaria [27-29]. On the down side, at two assessment sites, Kessounou and Malanville, the fabric integrity of more than half of the LLINs was compromised using the six months following distribution. The problem of physical damage could be addressed through better education [30] for correct hanging, care and repair of nets [31]. Solving the problem may require emphasis on less frequent washing of nets and more care when hanging and using the LLINs to increase the effective life of LLINs under field conditions, a critical modification, since national replacement of LLINs on any schedule less than every three years is, programmatically, highly unlikely.

LLINs are supposed to be effective even if torn [32,33], because of “repellency” associated with the insecticide. In this assessment, we applied a new method, which is faster than the WHO cone, does not require mosquitoes, is not destructive and easier to test statistically meaningful sample size, to capture and quantify insecticide residue on the LLINs surface. The GC method, used to estimate the proportion of nets with a surface insecticide level above the WHO threshold, uses a standardized sampling technique to sample the surface concentration of permethrin, and to compare the results to a GC threshold concentration of 2.73 μg permethrin/GC sample. The threshold concentration was estimated through testing LLINs that were previously evaluated by means of the WHO cone bio-assay test. Nets that exceeded the WHO test threshold value (a surface insecticide levels adequate to cause >80% mortality) as well as nets that were not (≤ 80% mortality) were identified and used to establish a GC cut off value that distinguishes the two groups. ROC analysis was used to compare bioassay and GC results. While potentially offering several advantages, the GC method as described here, was observed to have several limitations. The PE-150D LLINs technology incorporates molecules of permethrin in the nets fibers [34]. In theory the insecticide molecules migrate to the surface continuously replacing insecticide loss at the surface [35]. However, this regeneration process is not necessarily rapid and time (after washing) until completion of regeneration may be up to three weeks, during which the surface insecticide concentration, measured by the GC method, can be low [36]. This implies that the GC method may well have underestimated bio-efficacy. The multivariate analysis performed on the bio-efficacy data confirmed this observation because washing frequency and distance to water for washing were not associated with insecticide decay and suggested that other factor(s) was responsible of the low insecticide quantity observed in the ineffective LLINs. These results suggested that the GC method needs to be improved to assess the real bio-efficacy of these LLINs.

Our results support a conclusion that LLINs intervention, under field conditions [37], need to be strengthen by communication with community using LLIN to reduce removal practice and assure an effective impact of the distribution strategy. The results raise the question of whether or not greater emphasis on care and repair of nets at the household level could slow this process down. Additionally, LLIN manufacturers are working on the durability characteristics of the mesh used for manufacturing LLINs. A combination of cultural and net-related changes should be implemented and evaluated to improve LLIN effective life.

## Conclusion

LLINs were widely distributed by Benin NMCP in July 2011. We developed and tested a tool for monitoring the durability of the nets. After six months of use under field conditions, measures of integrity and bio-efficacy had dropped more quickly than anticipated. At present the WHO cone test appears to be the only reliable measure of net bio-efficacy for polyethylene-permethrin impregnated LLINs. The way in which the insecticide is incorporated into the LLIN fiber, and its migration to the surface during regeneration, may be responsible for confounding the GC results. If so, then the WHO cone test method may also be similarly affected. The results of other assessments could help us to better understand the effective operational life of this intervention.

## Competing interests

The authors declare that they have no competing interests.

## Authors’ contributions

RA: co-designed the study, designed experiments, coordinated field activities, collected and analyzed data, wrote and revised the paper; VG: mapping, participated in data collection, field activities and revised the paper; SH: participated in field activities and data collection; FO: assisted with the statistical analysis and participated in field activities and data collection; MG: technical assistance of the study and participated in design of the study; AM: designed the study, supervised field activities and revised the paper. All authors have read and approved the content of the submitted manuscript.
